# Aspirin Exposure and Mortality Risk among Prostate Cancer Patients: A Systematic Review and Meta-Analysis

**DOI:** 10.1155/2019/9379602

**Published:** 2019-04-03

**Authors:** Lai lai Fan, Cheng Peng Xie, Yi Ming Wu, Xi jie Gu, Ying he Chen, Yi jun Wang

**Affiliations:** Department of Urology, The Second Affiliated Hospital and Yuying Children's Hospital of Wenzhou Medical University, China

## Abstract

**Background:**

Prostate cancer (PCa) is the ninth most common cause of cancer death globally. Many studies have investigated aspirin exposure and mortality risk among PCa patients, returning inconsistent results. We conducted a comprehensive meta-analysis to explore the association between aspirin exposure and mortality risk among PCa patients and to investigate potential dose/duration/frequency-response relationships.

**Methods and Results:**

Studies published from 1980 to 2018 of PubMed and EMBASE databases were searched. We included 14 studies with 110,000 participants. Multivariate-adjusted odds ratios (ORs) were pooled using random-effect models. Potential dose/duration/frequency-response relationships were evaluated for aspirin exposure and prostate cancer-specific mortality (PCSM) risk. We did not detect an association between the highest aspirin exposure and mortality risk (PCSM of prediagnostic aspirin exposure, OR: 0.96, 95% confidence interval [CI]: 0.87-1. 07, I^2^= 0%; PCSM of postdiagnostic aspirin exposure, OR:0.92, 95% CI: 0.77-1.10, I^2^ = 56.9%; all-cause mortality [ACM] of prediagnostic aspirin exposure, OR: 0.96, 95% CI: 0.88-1.04, I^2^ = 9.4%; ACM of postdiagnostic aspirin exposure, OR: 0.95, 95% CI: 0.73-1.23, I^2^ = 88.9%). There was no significant dose/frequency-response association observed for aspirin exposure and PCSM risk. On duration-response analysis, we found that short-term postdiagnostic aspirin exposure (shorter than 2.5 years) increased the risk of PCSM.

**Conclusions:**

Our meta-analysis suggests that there is no association between aspirin exposure and PCSM risk. Nor is there an association between the highest aspirin exposure and ACM risk among PCa patients. More studies are needed for a further dose/duration/frequency-response meta-analysis.

## 1. Introduction

Prostate cancer (PCa) is the most commonly diagnosed cancer among men in over one-half of the countries of the world [1]. PCa is also the ninth most common cause of cancer death globally. It is estimated that there will be almost 1.3 million new cases of PCa and 359,000 associated deaths worldwide in 2018 [2]. Because of earlier diagnosis and improved treatment, death rates for PCa have been decreasing in many countries [3–5]. Epidemiologic studies have revealed many risk factors for PCa progression and death [6], possibly linked to a more westernized lifestyle, in combination with limited access to effective treatments [5, 7].

Aspirin as nonsteroidal anti-inflammatory drug (NSAID) that is widely used for preventing recurrent cardiovascular events [8] has been proposed as an anticancer agent to reduce cancer morbidity and mortality [9–12], especially for colorectal cancer. The molecular mechanism remains unclear; most researchers believe that the anticancer effect may be mediated through antithrombotic and anti-inflammation mechanisms via blockade of cyclooxygenase- (COX-) 1 and 2 isozymes, respectively [13]. In human PCa, the expression of both COX-1 and COX-2 is increased, possibly playing a role in the progression of the PCa [14, 15]. Many observational studies have examined whether aspirin affected PCa survival [11, 16–29]. However, the evidence from these studies has been inconsistent.

A meta-analysis had analyzed the association between aspirin exposure and mortality risk among PCa patients with an insignificant outcome [30]. This analysis used data published before 2016. However, they missed some important studies and included conference abstracts; they also committed errors of data extraction and did not explain heterogeneous source. Most important, they did not investigate potential dose/duration/frequency-response associations. To further explore the association between aspirin exposure and mortality risk among PCa patients, we included the latest studies and conducted a dose/duration/frequency-response meta-analysis to quantify the association between high dose/long term/high frequency exposure of aspirin and prostate cancer-specific mortality (PCSM) risk. To the best of our knowledge, this is the first study to investigate potential dose/duration/frequency-response associations between aspirin exposure and PCSM risk.

## 2. Methods

### 2.1. Search Strategy

We followed the meta-analysis of observational studies in epidemiology (MOOSE) guidelines [32]. In order to systematically retrieve studies describing the association between aspirin exposure and mortality risk, we first searched PubMed and EMBASE on April 10, 2018. We repeated the literature search on October 25, 2018, to verify that our research was based on latest data. References list of included studies and reviews were also checked. The search focused on four themes of subject terms and keywords: aspirin, nonsteroidal anti-inflammatory agents, prostate neoplasms, and mortality. The detailed search strategies are shown in the supplemental material (available here).

### 2.2. Study Selection

Literature eligibility was assessed by two investigators independently; discordant conclusions were resolved through discussion and consensus. Inclusion criteria were as follows: (1) the study was a cohort study or case-control study because of higher quality of evidence-based medical evidence; (2) reviews, case reports, letters, comments, and lectures were excluded; (3) the authors reported data from an original, peer-reviewed study; and (4) the exposure interest was aspirin exposure and the outcome was death, and the investigators reported multivariate-adjusted risk estimates with 95% confidence intervals (CIs). When articles had the same data source or included multiple publications, the articles of the most informative one or with the higher quality were included.

### 2.3. Data Extraction

The following information was extracted and transferred to specially designed forms from the included studies by two investigators independently: author name, publish year, study type, region, data source, age (mean age or age range), follow-up years or study period, number of participants with PCa, number of participants who died of PCa, death assessment method, aspirin assessment method, time of aspirin use, diagnostic method of PCa, T-stage of PCa, treatment of PCa, confounders adjustment, reference number, quality assessment, and corresponding risk estimates with 95% CIs on PCSM and all-cause mortality (ACM) of prediagnostic and postdiagnostic aspirin exposure. We took the highest dose of aspirin intake as the highest dose exposure. When the highest dose of aspirin was not available in the reports, we assigned the longest duration aspirin exposure as the highest dose exposure. For studies which provided a data of dose/duration/frequency-response analysis, risk estimates with 95% CIs for at least three quantitative categories of aspirin exposure were generated. If the required data was not readily available or clear from the published study, we attempted to collect relevant data by contacting the authors at least once.

We used the Newcastle-Ottawa Quality Assessment Scale (NOS) [33] to evaluate the quality of include studies. For nonrandomized studies, quality assessment includes the following aspects: selection, comparability, and exposure [34]. Different evaluation criteria were used for the cohort and case-control studies. The score of this scale is nine points, high quality is awarded bigger than or equal to seven points, four to six points is considered moderate quality, and poor quality is awarded less than or equal to three points. Poor quality studies would be excluded in the sensitivity analysis.

### 2.4. Data Synthesis and Analysis

We evaluated the association between aspirin exposure and mortality risk by using risk estimates. Hazard ratio (HR), relative ratio (RR), and standardized mortality ratio (SMR) values were considered reasonable approximations to odds ratio (OR) for the relatively rare outcome [35, 36]. Because studies report different exposure categories as tertiles, quartiles, and quintiles, study-specific OR for the highest dose of aspirin exposure was compared to the lowest dose of aspirin exposure. Forest plots were created to visually assess the mortality risk of the highest dose of aspirin exposure across studies. Cochrane Q statistic and the I^2^ statistic were used to test the heterogeneity across studies [37]. A p value < 0.10 was considered statistically significant for the Cochrane Q statistic. For I^2^ statistic, a value > 50% indicated a measure of heterogeneity. Pooled ORs were obtained using inverse-variance-weighted random-effects models of DerSimonian and Laird [38].

The method described by Greenland and Longnecker was used for the meta-analysis of the dose/duration/frequency-response association between aspirin exposure and PCSM risk [39, 40]. The method requires that the distributions of cases and controls, cumulative exposure, ORs, and 95% CIs for at least three quantitative exposure categories were known. When there were more than two studies reporting relevant data, the dose/duration/frequency-response meta-analyses were allowed. The median or mean dose/duration/frequency exposure in each category was used as the corresponding exposure. When there was no median or mean dose/duration/frequency exposure for each category in the reports, the midpoint of the upper and lower boundaries in each category was specified as average exposure. If the highest category was open ended, the midpoint of the category was set to 1.5 times the lower boundary. When the lowest category was open ended, the lower boundary was set to zero. Additionally, restricted cubic spine models with three (10, 50, and 90%) or four knots (5, 35, 65, and 95%) of the distribution of exposure were used to evaluate the potential linear or nonlinear associations between aspirin exposure and PCSM risk [41]. Linearity or nonlinearity relation was calculated by testing the null hypothesis that the coefficient of the second spline is equal to zero [42].

The impacts of study characteristics on the results were assessed by meta-regression of region, study type, number of participants, follow-up time, study quality, and mean age. Further subgroup analysis estimated the effects of region, study type, number of participants, follow-up time, study quality, mean age, adjusted for smoking, and adjusted for cardiovascular events. Remaining studies were reanalyzed following the omission of one study at a time to evaluate the stability and reliability of the results [43]. When the number of studies included was bigger than ten, the potential publication bias was examined by visual inspection of the funnel plot and the result of Egger regression asymmetry test [44].

Analyses were done with STATA version 14.1 (Stata Corp, College Station, Texas). A two-tailed p value < 0.05 was considered statistically significant.

## 3. Results

### 3.1. Literature Search

**Figure 1:**
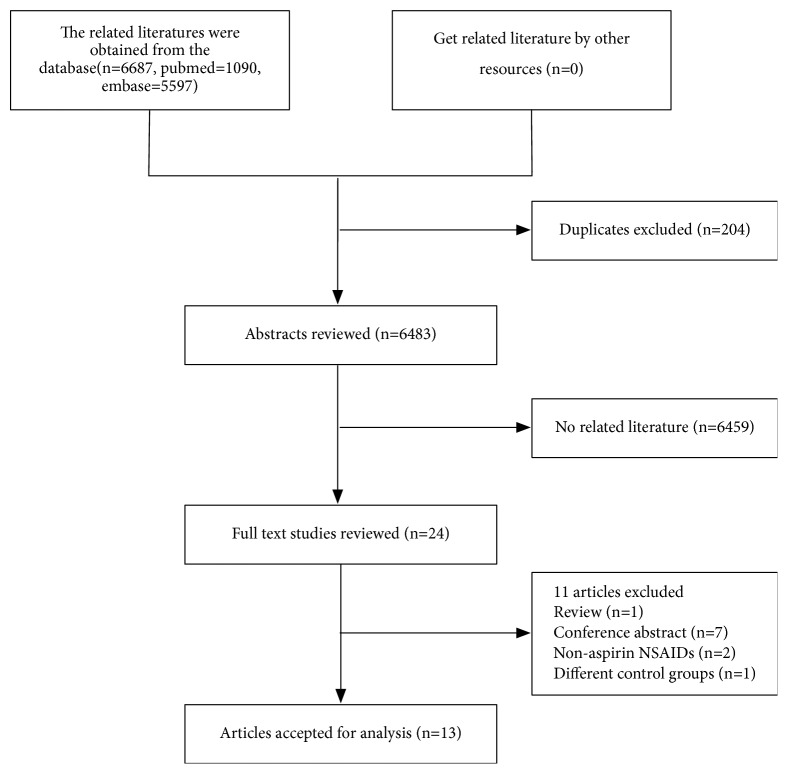
Flow diagram of systematic review of literature about aspirin exposure and mortality risk among prostate cancer patients.

Our initial search yielded 6,687 articles, of which we identified 204 duplicate articles. 24 articles were retained for further review after screening based on titles and abstracts. After detailed examination of these 24 full-text articles, 11 articles were excluded. 1 study [45] was excluded because the study was a review; 7 studies [31, 46–51] were excluded because they were conference abstracts; 2 studies were excluded because the exposure interests reported were nonaspirin NSAIDs [29, 52]; 1 study was excluded because the study used the normal population as a control group [11]. Ultimately, 13 articles [16–28] were included in our meta-analysis (Figure 1).

### 3.2. Study Characteristics

The characteristics of the included 13 articles are presented in Table 1. 1 article [16] included two studies of different data sources. Our meta-analysis included nearly 110 thousand participants with PCa, and we observed that nearly 10 thousand participants died of PCa. The participants of 9 studies [16–18, 21, 22, 26–28] were in America, and 5 [19, 20, 23–25] in Europe. 1 study [25] was designed as a case-control study; the remaining studies were designed as cohort studies. All the studies were published in or after 2012. 10 studies [16, 17, 19–21, 24–27] were graded as having high quality, and the remainder were of moderate quality; no study was evaluated as poor quality. The follow-up duration of cohort studies ranged from 3.25 to 9.3 years. The aspirin exposure assessment method was based on self-report in 6 studies [16, 22, 26–28], questionnaires in 2 studies [17, 21], and prescriptions in 5 studies [19, 20, 23–25]. The PCa death assessment method in most studies was based on death certificates. Diagnostic method of PCa was based on clinical or/and pathologic information in 8 studies [20–24, 26–28], medical records in 4 studies [16, 17, 19], and international statistical classification of diseases (ICD) codes in 1 study [25].

### 3.3. The Highest Dose of Aspirin Exposure and Mortality Risk

8 studies [16, 17, 19–21, 24, 25] examined prediagnostic aspirin exposure and 13 studies [16–23, 25–28] examined postdiagnostic aspirin exposure in relation to PCSM risk. 4 studies [16, 17, 20] examined prediagnostic aspirin exposure and 5 studies [16, 17, 20, 25] examined postdiagnostic aspirin exposure in relation to ACM risk. The outcome of prediagnostic aspirin exposure reported by Downer et al. [17] was excluded because they used the normal population as a control group. For ORs of the highest dose of aspirin exposure on PCSM, 1 study [24] reported a negative association of prediagnostic aspirin exposure, 1 study [20] reported a positive association of postdiagnostic aspirin exposure, and 2 studies [17, 28] reported a negative association of postdiagnostic aspirin exposure; the remaining studies reported that the ORs were not statistically different than 1.00. For ORs of the highest dose of aspirin exposure on ACM risk among PCa patients, 2 studies [16, 17] reported a negative association of postdiagnostic aspirin exposure and 2 studies [20, 25] reported a positive association of postdiagnostic aspirin exposure; the remaining studies reported that the ORs were not statistically different than 1.00.

**Figure 2:**
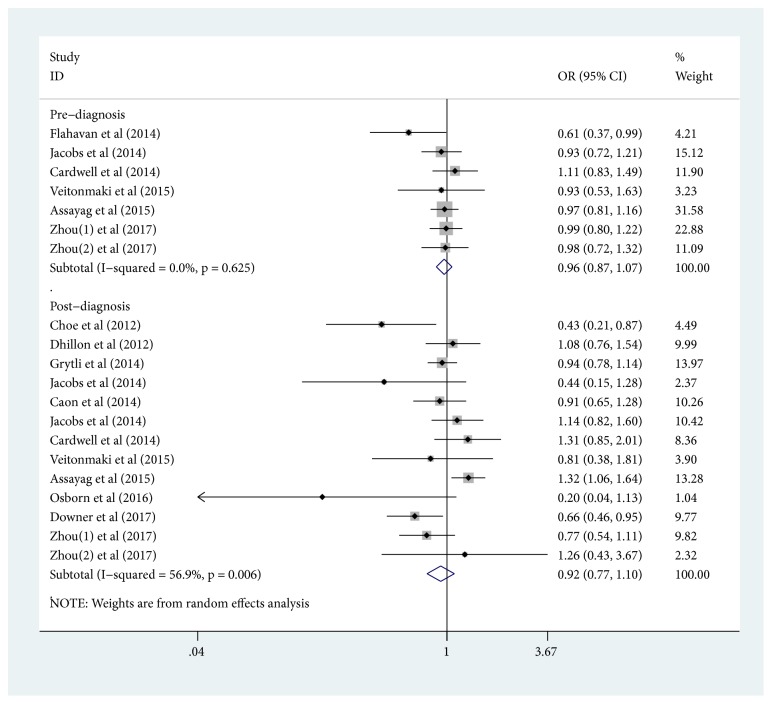
*Forest plots of aspirin exposure and prostate cancer-specific mortality risk*. (The squares and horizontal lines correspond to the study-specific OR and 95% CIs. The area of the squares reflects the study-specific weight. Weights are from random-effects analysis. The diamond represents the pooled OR and 95% CI.)

**Figure 3:**
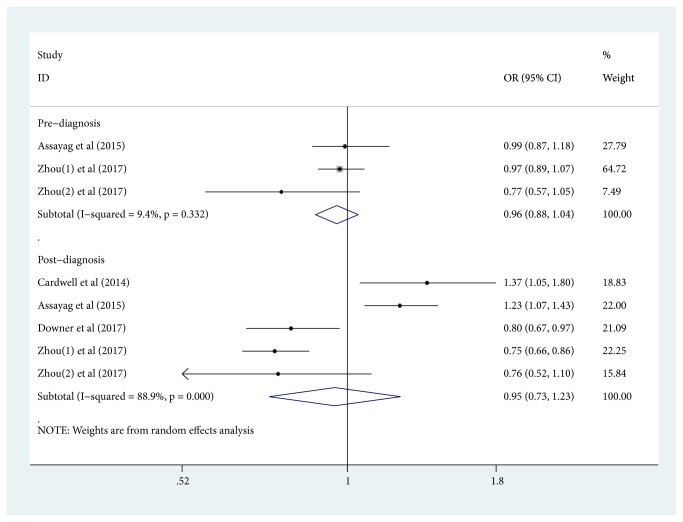
*Forest plots of aspirin exposure and all-cause mortality risk among prostate cancer patients*. (The squares and horizontal lines correspond to the study-specific OR and 95% CIs. The area of the squares reflects the study-specific weight. Weights are from random-effects analysis. The diamond represents the pooled OR and 95% CI.)

In the random-effects model, the pooled OR (95% CI) of the PCSM risk of prediagnostic aspirin exposure was 0.96(95% CI: 0.87-1. 07, Figure 2); the pooled OR (95% CI) of the PCSM risk of postdiagnostic aspirin exposure was 0.92(95% CI: 0.77-1. 10, Figure 2). We found an obvious heterogeneity (I^2^ =56.9%; p =0.006) in terms of outcome of postdiagnostic aspirin exposure. For ACM risk, the pooled OR (95% CI) of prediagnostic aspirin exposure from random-effects model was 0.96(95% CI: 0.88-1. 04, Figure 3); the pooled OR (95% CI) of postdiagnostic aspirin exposure from random-effects model was 0.95(95% CI: 0.73-1. 23, Figure 3). We also detected substantial heterogeneity (I^2^=88.9%; p≤0.001) in terms of outcome of postdiagnostic aspirin exposure.

**Figure 4:**
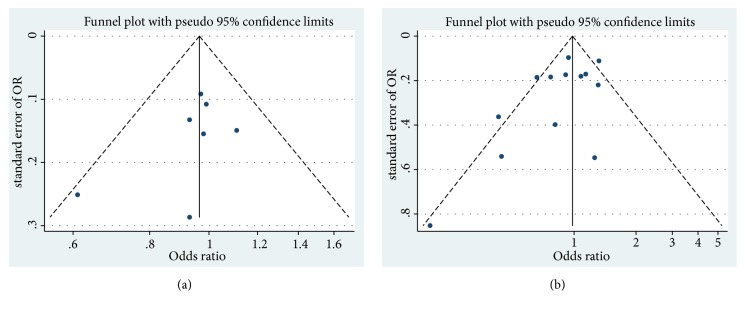
*Funnel plots for publication bias on the relationship between prostate cancer-specific mortality risk and prediagnostic aspirin exposure (a) and postdiagnostic aspirin exposure (b)*. (Circles represent identified studies.)

For PCSM risk, we detected a substantial heterogeneity of postdiagnostic aspirin exposure. To ascertain the heterogeneity of sources, we conducted a meta-regression analysis and the results were shown in the supplemental material. However, the results did not detect the source of the heterogeneity. Subgroup analyses were conducted by region, study type, number of participants, follow-up time, study quality, mean age, adjusted for smoking, and adjusted for cardiovascular events (Table 2). The subgroup of region (America: I^2^ =48.1%, 0R: 0.81, 95% CI: 0.65-1.03), participants (<5000: I^2^ =41.7%, 0R: 0.91, 95% CI: 0.74-1.11), age (<=68: I^2^ =0%, 0R: 0.51, 95% CI: 0.32-0.80), follow-up time (<=5: I^2^ =0%, 0R: 0.89, 95% CI: 0.76-1.05), quality (high: I^2^ =49.5%, 0R: 1.01, 95% CI: 0.84-1.21), adjusted for smoking (no: I^2^ =31.3%, 0R: 0.97, 95% CI: 0.79-1.18), and adjusted for cardiovascular events (no: I^2^ =37.4%, 0R: 0.96, 95% CI: 0.78-1.19) exhibited a decreases in heterogeneity. To further explore the sources of the heterogeneity, we performed the sensitivity analysis and found that the study by Assayag et al. was a major source of heterogeneity (from 42.1% to 56.9%). We omitted this study and performed the analysis again; the result remained insignificant (OR: 0.88, 95% CI: 0.75-1.05). The results of meta-regression and subgroup analyses did not indicate the source of heterogeneity, but the sensitivity analysis showed significant decreases of heterogeneity after excluding the study of Assayag et al. We found that the study of Assayag et al. reported the only positive result of PCSM risk on postdiagnostic aspirin exposure. Therefore, we speculated that the heterogeneity might derive from the study reported by Assayag et al. We found that the subgroup of less than or equal to 68 years old showed a significant negative association. Aspirin might have a little protective effect on younger patients with PCa. This result needed to be further verified because there were only 4 studies included. There was no publication bias according to the visual inspection of the funnel plot of prediagnostic aspirin exposure (Figure 4(a)) and postdiagnostic aspirin exposure (Figure 4(b)). The result of Egger's test of prediagnostic aspirin exposure (p = 0.276) and postdiagnostic aspirin exposure (p = 0.078) also showed no publication bias.

For ACM risk, we also detected substantial heterogeneity of postdiagnostic aspirin exposure. The sensitivity analysis of omitting one study at a time showed no substantial change in terms of results and heterogeneity. Because of the low number of studies that reported the aspirin exposure and ACM risk, subgroup and publication bias analyses were not pursued. Further studies are warranted.

### 3.4. Dose/Duration/Frequency-Response Meta-Analysis

For PCSM risk, 3 studies [21, 25, 27] examined dose of postdiagnostic aspirin exposure, 3 studies [17, 19, 20] examined duration of postdiagnostic aspirin exposure, and 3 studies [16, 21] examined frequency of both prediagnostic and postdiagnostic aspirin exposure. Every study contained relevant risk estimates with information for each exposure category reported. All studies were included in our meta-analysis. Because of a lack of data, we did not conduct a dose/duration/frequency-response meta-analysis on associations between aspirin exposure and ACM risk.

**Figure 5:**
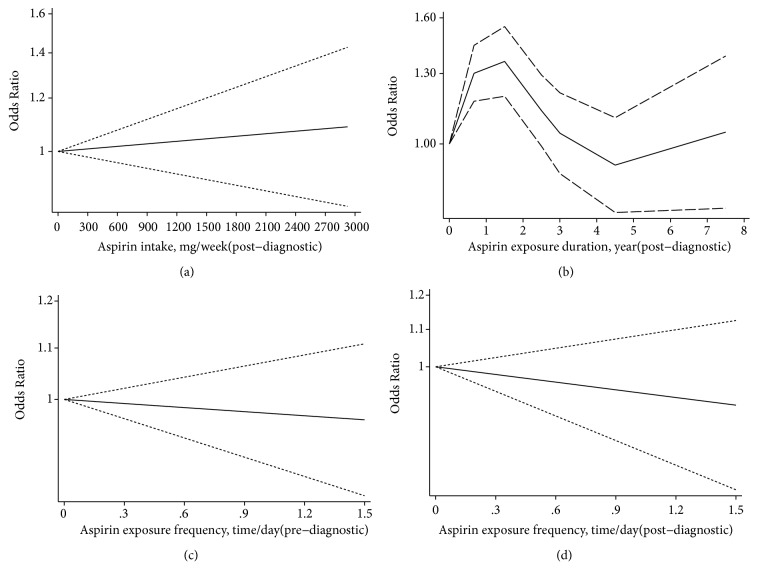
*(a) Dose-response relationship between postdiagnostic aspirin exposure and prostate cancer-specific mortality risk; (b) duration-response relationship between postdiagnostic aspirin exposure and prostate cancer-specific mortality risk; (c) frequency-response relationship between prediagnostic aspirin exposure and prostate cancer-specific mortality risk; (d) frequency-response relationship between postdiagnostic aspirin exposure and prostate cancer-specific mortality risk*. (The solid lines represent the linear/nonlinear trend. The dashed lines dashes represent the pointwise 95% confidence intervals for the linear trend.)

In the analysis of association between dose of postdiagnostic aspirin exposure and PCSM risk, we did not detect substantial heterogeneity (Q = 5.18, p =0.3937) and found a linearity association (p =0.7017). However, the result was not significant (Figure 5(a)). In the analysis of association between duration of postdiagnostic aspirin exposure and PCSM risk. We did not detect substantial heterogeneity (Q = 40.94, p ≤0.001) and found a nonlinearity association (p ≤0.001). The combined ORs of PCSM risk for 1.5, 2.5, and 3 years of duration exposure were 1.36 (95% CI: 1.19-1.55), 1.13 (95% CI: 0.99-1.29), and 1.04 (95% CI: 0.90-1.21), respectively (Figure 5(b)). Short-term aspirin exposure (shorter than 2.5 years) increased the risk of PCSM. The result needs to be further because of the limited number studies included. In the analysis of the association between frequency of prediagnostic aspirin exposure and PCSM risk, we did not detect substantial heterogeneity (Q = 1.89, p =0.7553) and found a linearity association (p =0.7956). The result was not significant (Figure 5(c)). In the analysis of association between frequency of postdiagnostic aspirin exposure and PCSM risk, we also did not detect substantial heterogeneity (Q = 2.07, p = 0.5327) and found a linearity association (p =0.5327). And the result was still not significant (Figure 5(d)).

## 4. Discussion

Many studies had investigated prediagnostic and postdiagnostic aspirin exposure with respect to mortality risk among PCa patients, with inconsistent results. The meta-analysis reported by Thakker et al. [30] had analyzed the association between aspirin exposure and mortality risk; they used the data published before 2016 and showed an insignificant outcome with substantial heterogeneity. They concluded that aspirin exposure was not associated with ACM and PCSM. However, they missed some important studies and included conference abstracts; they committed errors in data extraction and did not explain heterogeneous sources. Most important, they did not investigate potential dose/duration/frequency-response associations. The effect could have significant implications with respect to dose, frequency, and duration of aspirin use. To further explore the association between aspirin exposure and mortality risk, we updated the analysis and conducted a dose/duration/frequency-response meta-analysis to quantify the association between high dose/long term/high frequency exposure of aspirin and PCSM risk.

In this meta-analysis of 110,000 participants, we did not detect an association between the highest aspirin exposure and PCSM risk or any association regarding the highest aspirin exposure and ACM risk. The pooled ORs for PCSM of the highest postdiagnostic aspirin exposure were consistent in case-control and cohort studies. There was no significant dose-response association for dose of postdiagnostic aspirin exposure and PCSM risk. There was no significant frequency-response association for frequency of prediagnostic and postdiagnostic aspirin exposure and PCSM risk. In the meta-analysis of duration-response association, we found a nonlinearity association between duration of postdiagnostic aspirin exposure and PCSM risk. The result implied that short-term aspirin exposure (shorter than 2.5 years) increased the risk of PCSM. Indeed, premature discontinuation of drugs might mean disease progression; healthier men may continue to take aspirin. Androgen deprivation therapy had been associated with an increased risk of cardiovascular events [53, 54]. Health-conscious men with better prognosis might take aspirin earlier and longer for primary prevention. However, patients with chronic cardiovascular disease were more likely to be those long-term users of aspirin. The results require further verification for small studies. We did not conduct dose/duration/frequency-response meta-analysis of aspirin exposure and ACM risk because of lack of data. In the subgroup analysis, we found the subgroup of less than or equal to 68 years old had a significant negative association. Aspirin might have a small protective effect on younger patients with PCa, though age itself was a protective factor. This result needs to be further verified because there were only 4 studies included.

Whether aspirin protects against lethality of PCa is largely unknown. However, there have been various proposed mechanisms by which aspirin may improve oncologic outcomes. In colorectal cancer, clinical studies demonstrated that aspirin intake was associated with long-term incidence and mortality [55, 56]. Scholars who support this protective effect believe that platelets play a role in PCa metastasis by inducing angiogenesis, protecting tumor cells from immune surveillance, and promoting interactions between tumor cells and blood vessels [57–59]. Therefore, the antithrombotic effect of COX-1 inhibition of aspirin may impair PCa metastasis. The blockade of COX-2 could inhibit inflammation, suppress angiogenesis, and retain antimetastasis markers [60, 61]. The inhibition of COX-2 has inhibited PCa growth in both preclinical and human studies [62, 63]. Expression of both COX-1 and COX-2 was associated with increase in PCa [14, 15]. There are also COX-independent mechanisms that have been reported. However, the outcomes of our study did not accord with this view. Relative to inhibition of COX-1, aspirin has less potent COX-2 inhibitory action [64]. However, evidence reported recently is more likely to support the antitumor effect of COX-2 blockade [61, 65–67]. Therefore, a potent and selective inhibitor of COX-2 might represent an opportunity to augment current therapies. This is particularly of interest to patients with pain or undergoing radiation therapy where inflammation is a common side-effect. Further studies of selective inhibitors of COX-2 are needed.

Nevertheless, several limitations of our study should be acknowledged. First, this was a meta-analysis of observational studies; we could at best demonstrate an association but not a causal relationship. Second, heterogeneity was a potential problem when interpreting the results of our analysis. In analysis of PCSM risk and the highest postdiagnostic aspirin exposure, we found substantial heterogeneity, and we found the study by Assayag et al. was a major source of heterogeneity. The result remained insignificant after excluding this study. Third, the summary results might be influenced by the conversion of other measures to OR. Finally, the studies included in the dose/duration/frequency-response meta-analysis were limited; further studies are needed.

Our study also had several strengths: we performed a comprehensive systematic search for eligible studies; we conducted a dose/duration/frequency-response meta-analysis to quantify the association between high dose/long term/high frequency exposure of aspirin and PCSM risk; it was the first study to investigate potential dose/duration/frequency-response associations between aspirin exposure and PCSM risk; we included large enough numbers of participants; there was less possibility of publication bias; no substantial change in the results was found in the sensitivity analysis.

## 5. Conclusions

Our meta-analysis indicates that there is no association between aspirin exposure and PCSM risk. No association was found between highest aspirin exposure and ACM risk among PCa patients. More studies are needed to develop a further dose/duration/frequency-response meta-analysis.

## Figures and Tables

**Table 1:** Characteristics of studies included in the meta-analysis of aspirin exposure and mortality risk among prostate cancer patients.

Study, year	Study type	region	Data source	age	Follow-up time	Participants of PCa	Death of PCa	Death assessment	Aspirin assessment	use of aspirin	Diagnosis of PCa	T-stage of PCa	Treatment of PCa	Confounders adjustment	Reference number	OR of the highest dose exposure	Pattern score and OR	Quality assessment
Choe et al, 2012	retrospective cohort	US	CaPSURE	64	5.8	5955	193	death certificates, National Death Index and other sources	self-report	Post-diagnosis	clinical and pathologic information	I-IV	RT, RT+ADT, RP	NA	35	Post: 0.43(0.21,0.87)	NA	Selection: 3 Comparability: 1 Outcome: 2
Dhillon et al, 2012	retrospective cohort	US	the Health Professionals Follow-up Study	68.6	8.4	3986	265	National Death Index, postal system, and next of kin with virtually complete follow-up	self-report	Post-diagnosis	medical records and pathology reports	I-IIIa	PT, RT, Hormone, Watchful waiting, Others	age, period, family history, race, height, BMI, tomato sauce, vigorous physical activity, smoking, vitamin D, fish, red meat, CLD, total kcal, Gleason score, aspirin use before diagnosis, TNM stage, initial treatment	23	Post: 1.08(0.76,1.54)	Dose: Quartile 1: 1.0 Quartile 2: 1.12(0.72-1.72) Quartile 3: 1.05(0.62-1.80) Quartile 4: 1.08(0.76-1.54)	Selection: 3 Comparability: 2 Outcome: 2
Flahavan et al, 2014	retrospective cohort	Ireland	NCRI and GMS	50-80	5.5	2936	276	death certificates	prescriptions	Pre-diagnosis	pathologic information, ICD code	I–III	RP, PT, RT, ADT	age at diagnosis, tumor grade, tumor size, smoking status, co-morbidity score, year of incidence, pre-diagnostic statin exposure and receipt of radiation	20	Pre: 0.61(0.37,0.99)	NA	Selection: 3 Comparability: 2 Outcome: 2
Grytli et al, 2014	retrospective cohort	Norway	the Cancer Registry of Norway and the Norwegian Prescriptions Database	76.3	3.25	3165	NA	death certificates	prescriptions	Post-diagnosis	clinical and pathologic information	I-IV	ADT	age, PSA, Gleason score, T-stage, presence and type of metastases, performance status, and ADT initiated within 6 months after diagnosis	27	Post: 0.94(0.78,1.14)	NA	Selection: 3 Comparability: 2 Outcome: 1
Jacobs et al, 2014	retrospective cohort	US	the University of Texas Southwestern Medical Center	68	4.7	74	15	NA	self-report	Post-diagnosis	clinical and pathologic information	Ic-IIIb, unknown	RT, ADT	age, Gleason score, T-stage, pelvic irradiation, ADT, N-stage, aspirin use	24	Post: 0.44(0.15,1.28)	NA	Selection: 2 Comparability: 2 Outcome: 1
Caon et al, 2014	retrospective cohort	Canada	BCCA	70.3	8.4	3851	1098	death registry records	referring physician notes, consultation reports, self-report	Post-diagnosis	pathologic information	I-IV	RT	statin use, ASA use, age, ADT, PSA, T-stage, Charlson index, Gleason score	35	Post: 0.91(0.65,1.28)	NA	Selection: 3 Comparability: 2 Outcome: 2
Jacobs et al, 2014	retrospective cohort	US	CPS-II Nutrition Cohort	NA	Pre: 9.3 Post: 6.4	Pre: 8427 Post: 7118	Pre: 441 Post: 301	National Death Index	questionnaires	Pre-diagnosis and Post-diagnosis	clinical and pathologic information	I-IV	PT, RT, Cryosurgery, Hormone, Watchful waiting	age, race, calendar year of diagnosis, tumor extent, nodal involvement, Gleason score, initial treatment type, CLD, CVD, and pre-diagnosis PSA testing not leading to a PCa diagnosis.	19	Pre: 0.93(0.72,1.21) Post: 1.14(0.82,1.60)	Dose: Tertile 1: 1.00 Tertile 2: 0.85(0.61-1.19) Tertile 3: 1.14(0.82-1.60) Frequency(pre): Tertile 1: 1.00 Tertile 2: 1.03(0.81-1.32) Tertile 3: 0.92(0.72-1.17) Frequency(post): Tertile 1: 1.00 Tertile 2: 1.12(0.81-1.53) Tertile 3: 0.98(0.74-1.29)	Selection: 3 Comparability: 2 Outcome: 2
Cardwell et al, 2014	case–control	UK	NCDR, CPRD	NA	1998-2011	Pre: 5459 Post: 4715	Pre: 1371 Post: 1184	ONS death certificates	prescriptions	Pre-diagnosis and Post-diagnosis	ICD code	I-IV	RP, RT, CT, ADT, EST	grade, RP, CT, RT, ADT, EST, comorbidities and smoking	34	Pre: 1.11(0.83,1.49 Post: 1.31(0.85,2.01)	Dose: Quartile 1: 1.0 Quartile 2: 1.12(0.79-1.60) Quartile 3: 0.82(0.58-1.17) Quartile 4: 1.31(0.85-2.01)	Selection: 3 Comparability: 2 Outcome: 2
Veitonmaki et al, 2015	retrospective cohort	Finland	FinPCST	68	7.5	Pre: 6537 Post: 6537	Pre: 617 Post: 617	death certificates	prescriptions	Pre-diagnosis and Post-diagnosis	medical records	I-IV	PT, RT, Hormone, Watchful waiting	age, PCa stage and grade, type of treatment, CLD, anti-HPN drug, BPH drug and antidiabetic drug, use of NSAIDs before trial, other types of NSAIDs, PSA, cancer grade and stage.	23	Pre: 0.93(0.53,1.63) Post: 0.81(0.38,1.81)	Duration: Tertile 1: 1.00 Tertile 2: 0.54(0.27-1.10) Tertile 3: 0.31(0.12-0.78)	Selection: 3 Comparability: 2 Outcome: 2
Assayag et al, 2015	retrospective cohort	UK	the NCDR, CPRD, HES	71.3	5.4	Pre: NA Post: 11779	Pre: NA Post: 1793	ONS death certificates	prescriptions	Pre-diagnosis and Post-diagnosis	clinical information, ICD code	I-IV	PT, RT ADT, CT	age, year of entry, race, obesity, smoking status, alcohol use, socioeconomic status, anti-HPN drug, cardiovascular comorbidities, statins, aspirin, other APD, NSAIDs, 5a-reductase inhibitors, metformin, sulfonylureas, insulin, OADs, PSA, Gleason score and cancer treatments during first year after diagnosis	23	Pre: 0.97(0.81,1.16) Post: 1.32(1.06,1.64)	Duration: Quintile 1: 1.0 Quintile 2: 1.61(1.40-1.84) Quintile 3: 1.33(1.10-1.60) Quintile 4: 1.06(0.83-1.37) Quintile 5: 1.32(1.06-1.64)	Selection: 3 Comparability: 2 Outcome: 2
Osborn et al, 2016	retrospective cohort	US	the New York Harbor Department of Veterans Affairs	68	6.3	289	8	NA	physician documentation, the electronic medical record system	Post-diagnosis	NA	undergoing radiation	ADT, RT	age, ASA use, ADT, RT, clopidogrel or warfarin usage, NCCN risk group	20	Post: 0.20(0.04,1.13)	NA	Selection: 2 Comparability: 2 Outcome: 2
Downer et al, 2017	retrospective cohort	US	the Physicians' Health Study	71.5	NA	3277	407	death certificates, National Death Index, medical records and information from family	questionnaires	Pre-diagnosis and Post-diagnosis	self-reports and medical records	I-IV	RP, RT, others	age, calendar year of diagnosis, race, Charlson comorbidity index, BMI, smoking status, PSA, Gleason score, clinical stage, and primary treatment	28	Post 0.66(0.46,0.95)	Duration: Tertile 1: 1.00 Tertile 2: 0.70(0.50-0.97) Tertile 3: 0.66(0.46-0.95)	Selection: 3 Comparability: 2 Outcome: 2
Zhou [16] et al, 2017	retrospective cohort	US	NIH-AARP Diet and Health Study	>=55	Pre: 6 Post: 4	Pre: 19063 Post: 7574	Pre:709 Post:209	National Death Index	self-report	Pre-diagnosis and Post-diagnosis	medical records	I-IV	PT, RT, Hormone, RT+ Hormone	Gleason score, tumor stage, primary treatment, race, marital status, CVD, diabetes, BMI, smoking status, PCa screening, self-reported general health status, pre-diagnostic aspirin or non-aspirin NSAID use	30	Pre: 0.99(0.80,1.22) Post: 0.77(0.54,1.11)	Frequency(pre): Tertile 1: 1.00 Tertile 2: 0.95(0.78-1.15) Tertile 3: 0.99(0.80-1.22) Frequency(post): Tertile 1: 1.00 Tertile 2: 0.87(0.60-1.27) Tertile 3: 0.77(0.54-1.11)	Selection: 3 Comparability: 2 Outcome: 2
Zhou [31] et al, 2017	retrospective cohort	US	PLCO Cancer Screening Trial	>=55	Pre: 5 Post: 5	Pre: 7827 Post: 4012	Pre:266 Post:35	death certificates	self-report	Pre-diagnosis and Post-diagnosis	medical records	I-IV	PT, RT, Hormone, RT+ Hormone	Gleason score, tumor stage, primary treatment, race, marital status, CVD, diabetes, BMI, smoking status, PCa screening, self-reported general health status, pre-diagnostic aspirin or non-aspirin NSAID use	30	Pre: 0.98(0.72,1.32) Post: 1.26(0.43,3.67)	Frequency(pre): Tertile 1: 1.00 Tertile 2: 1.15(0.85-1.55) Tertile 3: 0.98(0.72-1.32) Frequency(post): Tertile 1: 1.00 Tertile 2: 1.52(0.53-4.33) Tertile 3: 1.26(0.43-3.67)	Selection: 3 Comparability: 2 Outcome: 2

PCa: prostate cancer; USDA: the United States Department of Agriculture; ATBC: Alpha-Tocopherol Beta-Carotene Cancer Prevention Study; BLSA: Baltimore Longitudinal Study of Aging; WNYDS: Western New York Diet Study; NECSS: National Enhanced Cancer Surveillance System; PLCO: Prostate, Lung, Colorectal, and Ovarian Cancer Screening Trial; MDC: Malmo Diet and Cancer; EECC: Environmental Epidemiology of Cancer in Cordoba; DVAMC: Durham Veterans Affairs Medical Center; FHS: Framingham Heart Study; EPIC: European Prospective Investigation into Cancer and Nutrition; NSHD: National Survey of Health and Development; ProtecT: Prostate testing for cancer and Treatment; FFQ: food frequency questionnaire; ICD: international statistical classification of diseases; BMI: body mass index; PSA: prostate-specific antigen.

**Table 2:** Subgroup analyses of the highest post-diagnostic aspirin exposure and prostate cancer-specific mortality risk.

Group	OR(95%CI)	Number of studies	I^2^ (%)	P_(heterogeneity)_
Region				
America	0.81(0.65,1.03)	9	48.1	0.052
Europe	1.12(0.88,1.41)	4	54.1	0.088

Study type				
case-control	1.31(0.85,2.01)	1	NA	NA
cohort	0.89(0.74,1.07)	12	57.8	0.006

Participants				
	0.91(0.74,1.11)	8	41.7	0.100
	0.92(0.65,1.28)	5	70.8	0.008

Age				
	0.51(0.32,0.80)	4	0	0.417
	0.98(0.79,1.21)	5	67	0.017

Follow-up time				
	0.89(0.76,1.05)	4	0	0.392
	0.96(0.74,1.24)	7	60.5	0.019

Quality^*∗*^				
moderate	0.55(0.29,1.05)	4	66.4	0.030
high	1.01(0.84,1.21)	9	49.5	0.045

Adjusted for smoking				
yes	0.96(0.71,1.31)	5	70.3	0.009
no	0.97(0.79,1.18)	7	31.3	0.189

Adjusted for cardiovascular events				
yes	0.96(0.72,1.27)	6	64.6	0.015
no	0.96(0.78,1.19)	6	37.4	0.157

^*∗*^A total score of 4-6 was considered moderate quality, and 7-9 was deemed high quality.

## Supplementary Materials

Supplementary Materials
* S1*: search strategies in PubMed and EMBASE.* S2*: meta-regression of the highest postdiagnostic aspirin exposure and prostate cancer-specific mortality risk.
