# Supplementary material for: Aspirin Exposure and Mortality Risk among Prostate Cancer Patients: A Systematic Review and Meta-Analysis
Source: Biomed Res Int. 2019 Apr 3;2019:9379602. doi: 10.1155/2019/9379602 (PMC6470443; doi:10.1155/2019/9379602)
Supplement: Supplementary Materials — S1: search strategies in PubMed and EMBASE. S2: meta-regression of the highest postdiagnostic aspirin exposure and prostate cancer-specific mortality risk. [file 9379602.f1.docx]

**Search strategies in PubMed and EMBASE**

**Search strategies in Pubmed :**

1. (((((((((((((Aspirin[Title/Abstract] OR flufenamic acid[Title/Abstract] OR ibuprofen[Title/Abstract] OR ketoprofen[Title/Abstract] OR sulindac[Title/Abstract] OR flurbiprofen[Title/Abstract] OR diclofenac[Title/Abstract] OR naproxen[Title/Abstract] OR tenoxicam[Title/Abstract] OR fenoprofen[Title/Abstract] OR oxaprozin[Title/Abstract] OR mefenamic acid[Title/Abstract] OR tolfenamic acid[Title/Abstract] OR piroxicam[Title/Abstract] OR indomethacin[Title/Abstract] OR indometacin[Title/Abstract] OR feprazone[Title/Abstract] OR phenylbutazone[Title/Abstract] OR isoxicam[Title/Abstract] OR meclofenamate[Title/Abstract] OR ketorolac[Title/Abstract] OR droxicam[Title/Abstract] OR lornoxicam[Title/Abstract] OR etoricoxib[Title/Abstract] OR etodolac[Title/Abstract] OR celecoxib[Title/Abstract] OR meloxicam[Title/Abstract] OR lumiracoxib[Title/Abstract] OR nimesulide[Title/Abstract])) OR Cyclooxygenase 2 inhibitor[Title/Abstract]) OR cox 2 inhibitor[Title/Abstract])) OR NSAIDs[Title/Abstract])) OR ("Cyclooxygenase Inhibitors"[Mesh] OR "Cyclooxygenase 2 Inhibitors"[Mesh])) OR "Aspirin"[Mesh]) OR ("Anti-Inflammatory Agents"[Mesh] OR "Anti-Inflammatory Agents, Non-Steroidal"[Mesh])))
2. ((((((prostat*[Title/Abstract]) AND (cancer*[Title/Abstract] OR neoplasms*[Title/Abstract] OR carcinoma*[Title/Abstract] OR tumour*[Title/Abstract]))) OR "Prostatic Neoplasms"[Mesh]) OR "Prostatic Intraepithelial Neoplasia"[Mesh]) OR Prostatic Intraepithelial Neoplasia[Title/Abstract])))
3. #1 And #2

**Search strategies in Emabase:**

1. 'acetylsalicylic acid':ab,ti OR 'flufenamic acid':ab,ti OR 'ibuprofen':ab,ti OR 'ketoprofen':ab,ti OR 'sulindac':ab,ti OR 'flurbiprofen':ab,ti OR 'diclofenac':ab,ti OR 'naproxen':ab,ti OR 'tenoxicam':ab,ti OR 'fenoprofen':ab,ti OR 'oxaprozin':ab,ti OR 'mefenamic acid':ab,ti OR 'tolfenamic acid':ab,ti OR 'piroxicam':ab,ti OR 'indometacin':ab,ti OR 'feprazone':ab,ti OR 'phenylbutazone':ab,ti OR 'isoxicam':ab,ti OR 'meclofenamic acid':ab,ti OR 'ketorolac':ab,ti OR 'droxicam':ab,ti OR 'lornoxicam':ab,ti OR 'etoricoxib':ab,ti OR 'etodolac':ab,ti OR 'celecoxib':ab,ti OR 'meloxicam':ab,ti OR 'lumiracoxib':ab,ti OR 'nimesulide':ab,ti OR 'cyclooxygenase 2 inhibitor':ab,ti OR'cyclooxygenase 2 inhibitor'/exp OR 'prostaglandin synthase inhibitor'/exp OR 'aspirin'/exp OR 'antiinflammatory agent'/exp OR 'nonsteroid antiinflammatory agent'/exp
2. 'prostatic neoplasms'/exp OR 'prostatic intraepithelial neoplasia'/exp OR 'prostat*':ab,ti AND ('cancer*':ab,ti OR 'neoplasms*':ab,ti OR 'carcinoma*':ab,ti OR 'tumour*':ab,ti) OR 'prostatic intraepithelial neoplasia':ab,ti
3. 'mortality':ab,ti OR 'death':ab,ti OR 'survival':ab,ti
4. #1 And #2 And #3

| Group | P |
| --- | --- |
| Region | 0.118 |
| Study type | 0.299 |
| Participants | 0.099 |
| Follow-up time | 0.760 |
| Quality | 0.084 |
| age | 0.232 |

**Meta-regression of the highest post-diagnostic aspirin exposure and prostate cancer-specific mortality risk.**
